# Secretion of tumoricidal human tumor necrosis factor-related apoptosis-inducing ligand (TRAIL) by recombinant *Lactococcus lactis*: optimization of in vitro synthesis conditions

**DOI:** 10.1186/s12934-018-1028-2

**Published:** 2018-11-16

**Authors:** Katarzyna Ciaćma, Jerzy Więckiewicz, Sylwia Kędracka-Krok, Magdalena Kurtyka, Małgorzata Stec, Maciej Siedlar, Jarek Baran

**Affiliations:** 1 Department of Clinical Immunology, Institute of Pediatrics, Jagiellonian University Medical College, Wielicka str. 265, 30-663 Kraków, Poland; 2 Department of Physical Biochemistry, Faculty of Biochemistry, Biophysics and Biotechnology, Jagiellonian University, Kraków, Poland; 3 Proteomics and Mass Spectrometry Laboratory, Malopolska Centre of Biotechnology, Jagiellonian University, Kraków, Poland

**Keywords:** TRAIL, *Lactococcus lactis*, Nisin Controlled Gene Expression System, Colorectal cancer

## Abstract

**Background:**

Tumor necrosis factor-related apoptosis-inducing ligand (TRAIL) selectively eliminates tumor cells. However, the short biological half-life of this molecule limits its potential use in the clinic. Our aim was to construct a recombinant strain of nonpathogenic *Lactococcus lactis* bacteria as a vector for effective and prolonged human TRAIL production. Herein, we examined the expression and secretion conditions leading to the production of biologically active protein in vitro.

**Results:**

The human soluble TRAIL-cDNA (hsTRAIL-cDNA) with optimized codons was designed to fit the codon usage pattern (codon bias) of the *L. lactis* host. This cDNA construct was synthesized and cloned in *lactococcal* plasmid secretion vector pNZ8124 under the control of the nisin-induced PnisA promoter. The pNZ8124-hsTRAIL plasmid vector was transformed into the *L. lactis* NZ9000 host strain cells by electroporation. Secretion of the protein occurred at the neutral pH during induction, with optimized concentration of the inducer and presence of serine proteases inhibitor. Using Western blotting and amino acid sequencing method we found that TRAIL was secreted in two forms, as visualized by the presence of two distinct molecular size bands, both deprived of the usp45 protein, the bacterial signal peptide. By the use of MTS assay we were able to prove that hsTRAIL present in supernatant from *L. lactis* (hsTRAIL+) broth culture was cytotoxic to human HCT116 colon cancer cells but not to normal human fibroblasts. Flow cytometry analysis revealed TRAIL-induced apoptosis of cancer cells.

**Conclusions:**

We designed recombinant *L. lactis* bacteria, which efficiently produce biologically active, anti-tumorigenic human TRAIL in vitro. Further studies in tumor-bearing NOD-SCID mice will reveal whether the TRAIL-secreting *L. lactis* bacteria can be used as a safe carrier of this protein, capable of inducing effective elimination of human colon cancer cells in vivo.

## Background

Colorectal cancer is one of the most common gastrointestinal cancers worldwide [1]. The standard treatment, which includes surgery followed by chemotherapy often induces drug resistance of tumor cells and tumor relapse. Therefore the need for a novel form of a more effective treatment is still urgent [2]. Tumor necrosis factor-related apoptosis-inducing ligand [3, 4] (TRAIL, other names: TNFSF10; CD253; Apo-2L; TNLG6A [5]), is a protein belonging to the TNF-superfamily [6] and has been shown in in vitro and in vivo models to induce apoptosis of various types of cancer cells while sparing normal ones [7]. TRAIL may act as a trans-membrane protein or can be cleaved from the cell surface by cathepsin E to form a soluble ligand. Both forms of TRAIL are biologically active and their interactions with specific death receptors TRAIL-R1 (DR4) and TRAIL-R2 (DR5) induce apoptosis of cancer cells upon activation of caspase-8 [4, 8]. The only form of human TRAIL approved currently for the use in clinical trials is its recombinant soluble and untagged version—dulanermin (Apo2L.0, AMG-951), developed by Genentech [9]. Dulanermin has been tested in combination with different cytostatic drugs in phase I-clinical studies concerning colorectal cancer patients, in which it proved to be safe, however, with limited activity because of its low pharmacokinetic profile [10–12]. Therefore, recent research on the new TRAIL formulations was focused on enhancing its bioactivity for cancer treatment and increasing its stability in humans [10].

*Lactococcus lactis* is a non-pathogenic, Gram-positive bacterium, for years widely used in dairying. Because of its safety and potential for direct secretion (monolayer cell wall) of heterologous proteins into the extracellular environment [13], it gained an interest as a host for production of recombinant proteins, including therapeutics [14], especially after the development of the Nisin Controlled Gene Expression System (NICE^®^). This solution guaranteed, among others, tightly controlled, endotoxin-free and without formation of inclusion bodies, food-grade expression of proteins [15]. The role of *L. lactis* bacteria in potential treatment of colorectal cancer is worth noticing, since lactic acid bacteria (LAB) are common microflora of the gut’s ecosystem and, therefore, present the possibility of introducing therapeutic proteins locally. Recently, Zhang et al. showed the ability of recombinant *L. lactis* NZ9000 strain to produce KiSS-1 peptide, a cancer suppressor factor, which inhibited proliferation and migration of human colon cancer HT-29 cells in vitro [16]. In our study we propose *L. lactis* bacteria as the host for an efficient expression of a secretory bioactive form of human TRAIL (human soluble TRAIL; hsTRAIL) under the control of the nisin-induced PnisA promoter, which would enable elimination of human colorectal cancer HCT116 cells in vitro and in vivo. In this paper we focused on optimization of the culture and secretion conditions for recombinant *L. lactis* strain, leading to the production of biologically active protein. To the best of our knowledge, this is the first study providing evidence that genetically engineered *L. lactis* bacteria, harbouring a plasmid with hsTRAIL-cDNA, may be an applicable carrier for efficient expression, secretion and safe delivery of bioactive hsTRAIL for elimination in vitro of colorectal cancer cells.

## Results

### *Lactococcus lactis* (hsTRAIL+) bacteria require specific conditions for growth and efficient expression of recombinant hsTRAIL

**Fig. 1:**
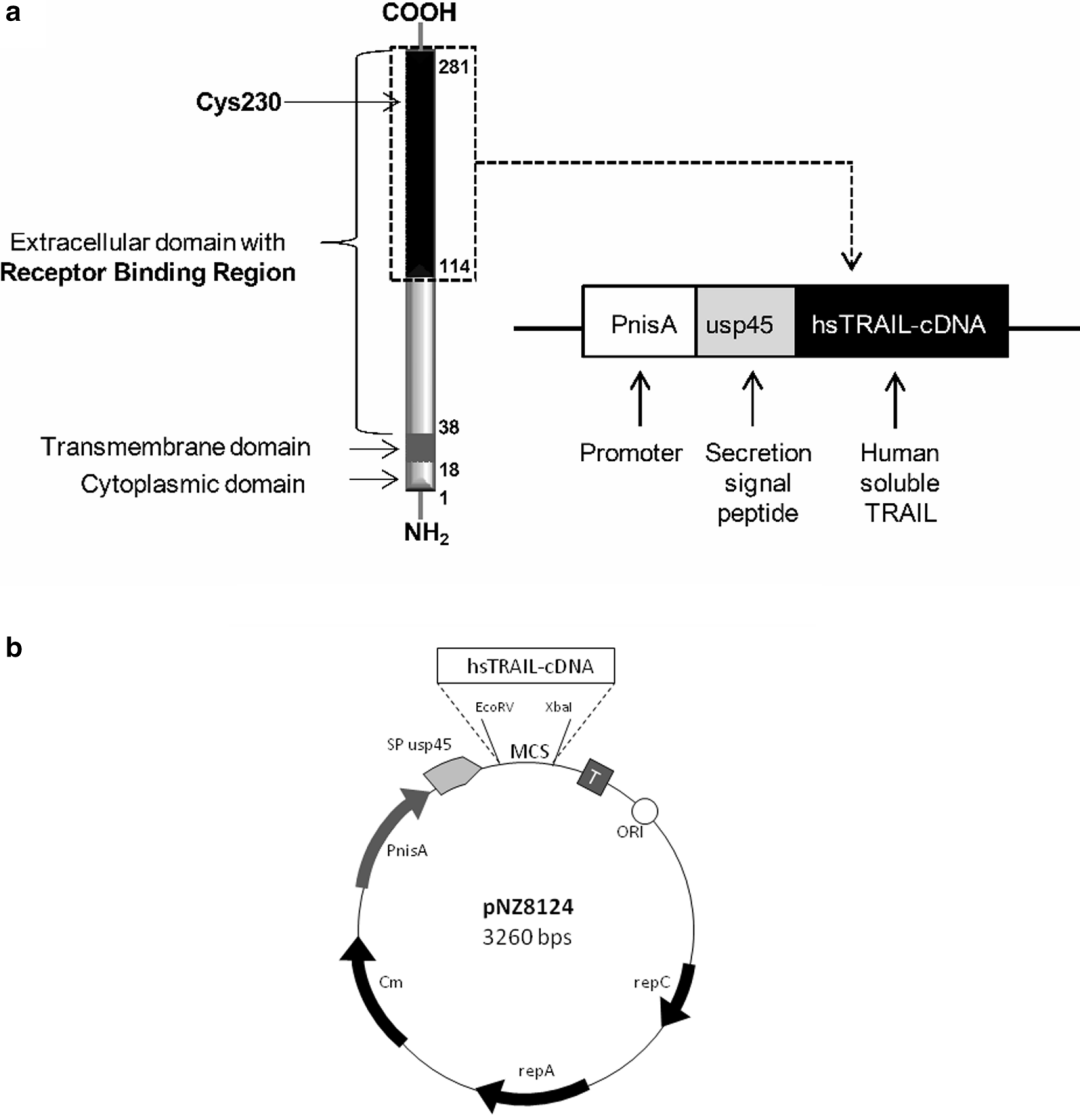
Schematic presentation of the TRAIL protein structure and map of the pNZ8124 secretion vector with expression cassette for controlled hsTRAIL production. **a** Left—TRAIL is a type II transmembrane protein composed of three major parts: an extracellular TNF-like domain (38–281 aa) with receptor binding region (114–281 aa) and cysteine residue (Cys230), which together with zinc ion are essential for the interaction between three TRAIL-molecules and formation of active TRAIL-homotrimer; a transmembrane domain (18–38 aa) and small cytoplasmic domain (1–18 aa). Transmembrane TRAIL-ligand might be cleaved at an extracellular stalk and released as a soluble molecule. Both forms of TRAIL-ligand (membrane-bound and soluble) are active. Modified from [42]. Right—codon-optimized hsTRAIL-cDNA sequence (coding the region 114–281 aa of human TRAIL) was inserted into pNZ8124 vector plasmid and transformed into *L. lactis* NZ9000 host strain cells via electroporation. The hsTRAIL expression was based on the Nisin Controlled Gene Expression System (NICE^®^) with nisin as the inducer. Protein secretion into the medium was obtained by the presence of sequence for signal peptide of *L. lactis* usp45 gene (*usp45*) downstream from promoter region (PnisA). **b** The pNZ8124 vector contains region for the nisin-inducible promoter (PnisA), an origin of replication sequence (ORI), two genes for the replication proteins (repA, repC), the transcription termination sequence (T) and the gene for the resistance to chloramphenicol (Cm) as the selection marker. The cDNA for hsTRAIL was inserted downstream of the promoter region and the signal sequence for *usp45* gene (SP usp45) on the plasmid, between EcoRV and XbaI sites from the multicloning site sequence (MCS). The secretion vector with hsTRAIL-cDNA insert has no any tag sequence added. The vector map is not drawn in scale

**Fig. 2:**
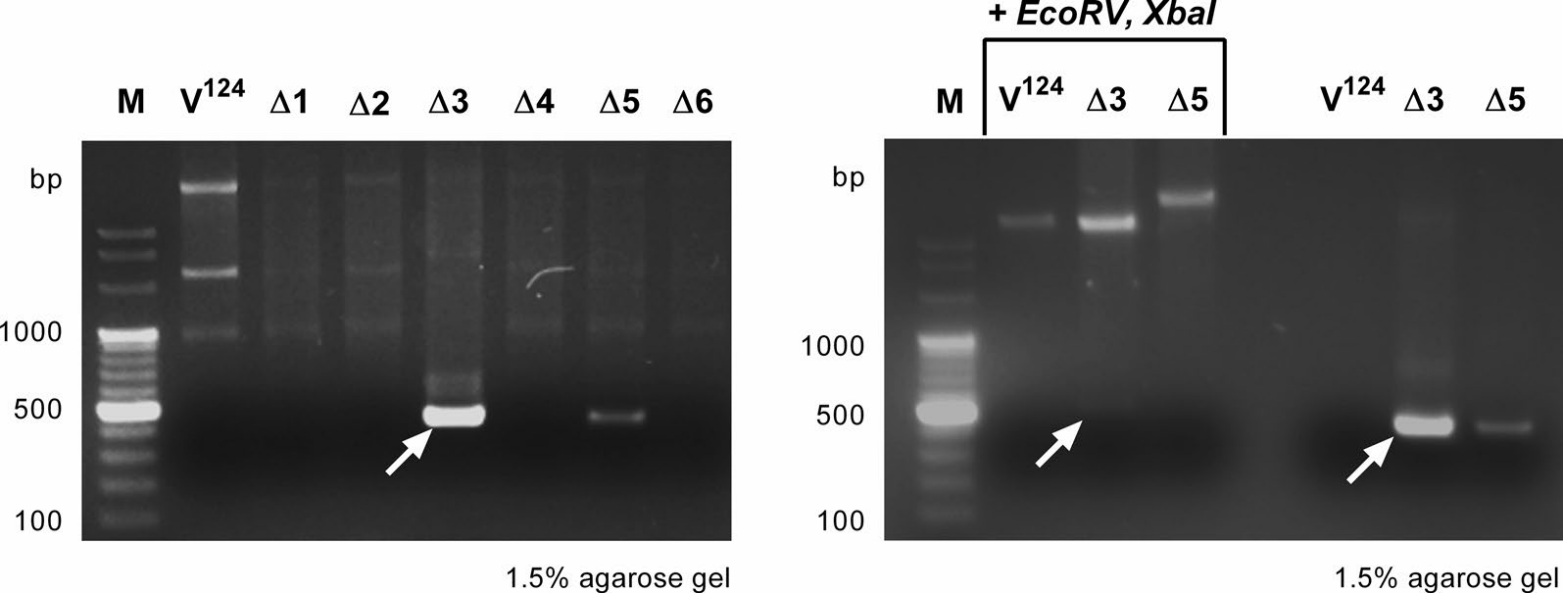
Selection of *L. lactis* (hsTRAIL+) clones. Left—results of DNA electrophoretic separation of PCR reaction products. As templates for PCR reaction, plasmids isolated from *L. lactis* recombinant clones (∆1–∆6), were used. White arrow- sequence for hsTRAIL (438 bp) presents in selected *L. lactis* clone ∆3. Right—results from DNA electrophoresis for hsTRAIL-insert after digestion of isolated plasmids with *EcoRV* and *XbaI* (on the left) and after PCR reaction with digested inserts used as the templates (on the right). White arrow—sequence for hsTRAIL (438 bp) presents in selected *L. lactis* clone ∆3. M-DNA molecular weight marker; V^124^—plasmid vector pNZ8124 without hsTRAIL-insert (negative control)

**Fig. 3:**
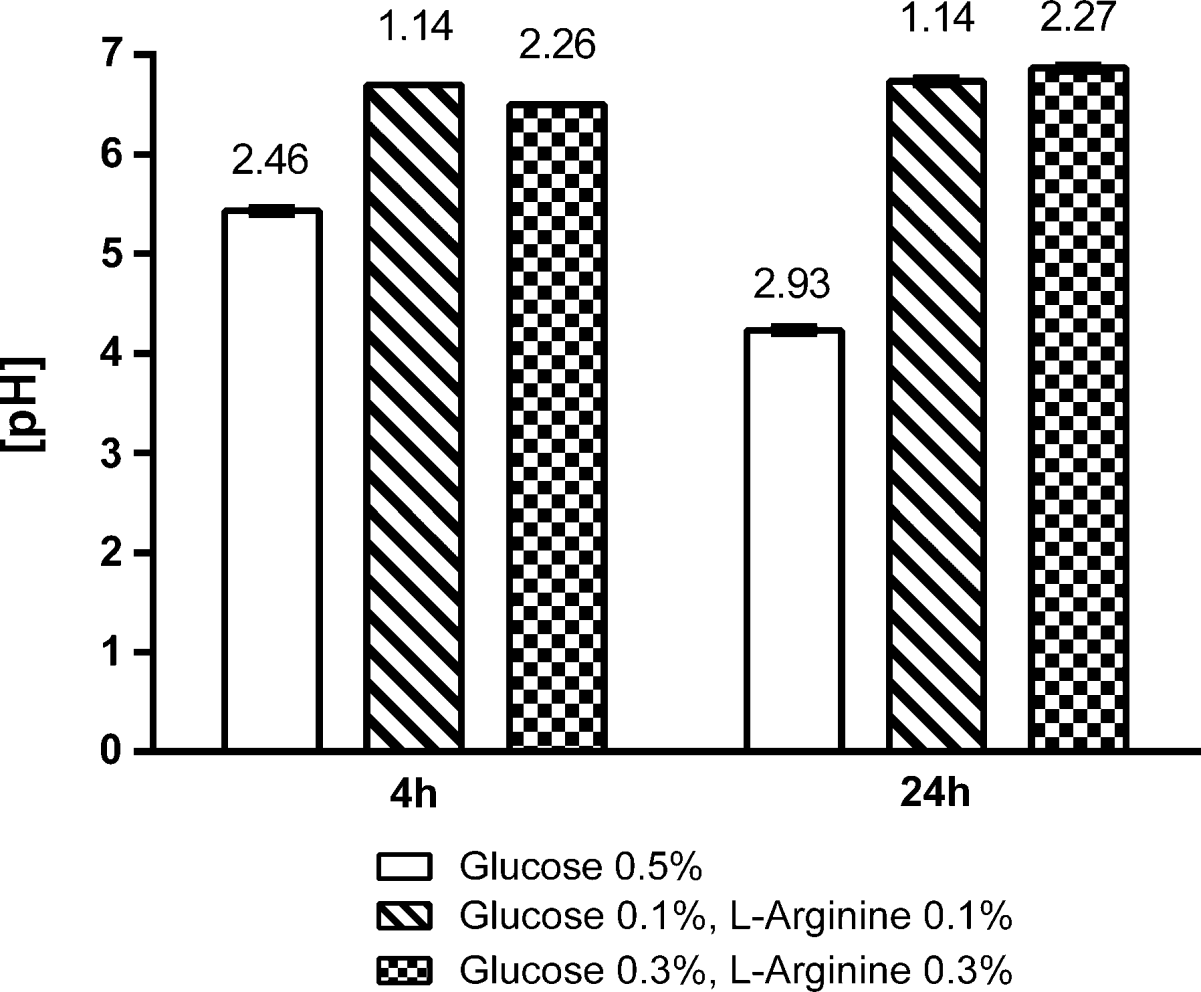
Optimization of growth conditions for *L. lactis* (hsTRAIL+) bacteria. *L. lactis* (hsTRAIL+) bacteria were cultured in M17 broth medium supplemented with chloramphenicol (10 µg/ml) and different concentration of glucose or/and l-arginine, as the source of energy. After 4 and 24 h of culture, pH (y-axis) and optical density at 600 nm (OD600; indicated by the numbers) were measured. For further experiments, the combination of glucose and l-arginine at 0.3% both, was selected. Experiment was performed three times, each in triplicates and mean values ± SD are presented

For the most effective expression of recombinant hsTRAIL in *L. lactis* bacteria, we designed a synthetic hsTRAIL-cDNA with optimized codons to fit the codon usage pattern (codon bias) of *L. lactis* as the host [18–20]. To ensure an efficient secretion of hsTRAIL by recombinant bacteria, we used a plasmid vector pNZ8124 containing the signal sequence of the *lactococcal* major secreted protein usp45, located downstream of the nisin-inducible PnisA promoter (Fig. 1). The two-step screening of *L. lactis* clones after electroporation revealed clone number 3 (Fig. 2), as the one harbouring pNZ8124 plasmid with hsTRAIL-cDNA. This clone was designated as *L. lacti*s (hsTRAIL+) and selected for further studies. Subsequently, we optimized the culture conditions for hsTRAIL-producing *L. lactis* clone. For this reason we determined how the combination of glucose and l-arginine, as sources of energy may affect both density and pH of *L. lactis* (hsTRAIL+) culture after 4 and 24 h of their static growth. We observed that only the combination of 0.3% glucose and 0.3% of l-arginine enabled satisfactory bacterial culture density (OD_600_) and neutral pH over time (Fig. 3). This combination of glucose and l-arginine concentrations was selected for further studies.

**Fig. 4:**
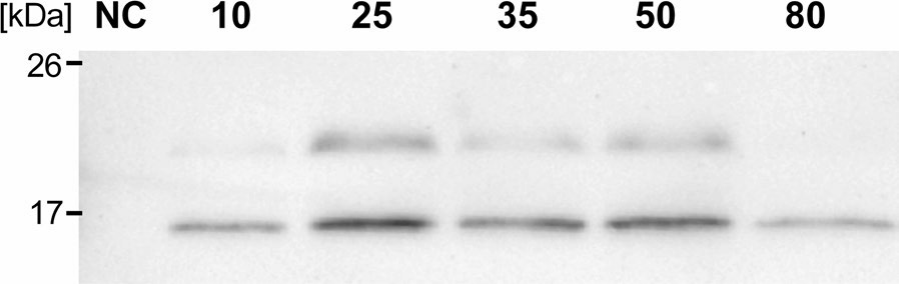
Optimization of hsTRAIL expression by *L. lactis* (hsTRAIL+) using the Nisin Controlled Gene Expression System (NICE^®^). TRAIL-protein was precipitated from supernatants from broth cultures of *L. lactis* (hsTRAIL+), induced for hsTRAIL expression with different concentrations of nisin. The samples were subjected to SDS-PAGE electrophoresis and Western blot analysis. As a negative control (NC) supernatant from non-induced broth cultures of *L. lactis* (hsTRAIL+) was used in parallel. For further experiments, the nisin concentration of 25 ng/ml was selected. NC—negative control; 10–80 (ng/ml)—concentration of nisin used for the induction. Data from one representative experiment out of three performed are shown

One of the critical steps in the NICE^®^ expression system is the amount of nisin added as the inducer of protein expression [15]. In the study performed by Miereau et al., the authors showed a strong correlation between the cell density at the time of induction and the amount of nisin required to obtain maximal induction rate [17]. Therefore, we also examined the effect of different concentrations of nisin on hsTRAIL expression by *L. lactis* (hsTRAIL+) and found 25 ng/ml as the most effective (Fig. 4). This concentration was used in further studies.

### *L. lactis* (hsTRAIL+) bacteria secrete hsTRAIL into the culture supernatant

**Fig. 5:**
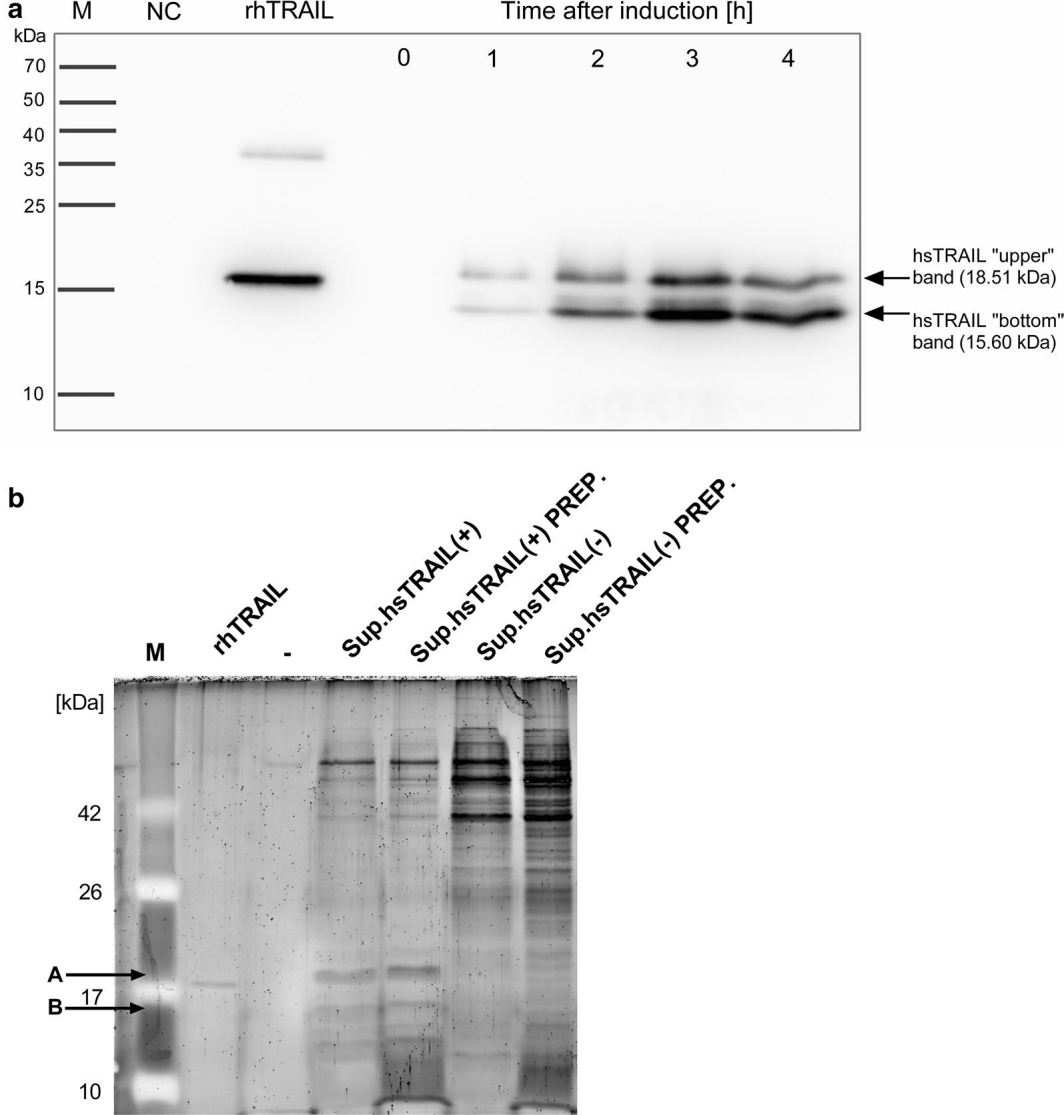
Detection of hsTRAIL protein production and secretion by *L. lactis* (hsTRAIL+). **a** hsTRAIL was detected in supernatants from broth cultures of *L. lactis* (hsTRAIL+) by Western blot and appeared in two bands indicating 160 aa (122–281) hsTRAIL molecule without usp45 leader peptide and devoids of 9 amino acids from N-terminus (upper band) and more truncated hsTRAIL ligand with 132 aa (150–281; lower band). Secretion of hsTRAIL started within the first hour after beginning of the induction with nisin (25 ng/ml). M—molecular weight marker; rhTRAIL (PeproTech, London, UK)—recombinant human TRAIL used as positive control; NC—protein sample precipitated from supernatant of *L. lactis* (hsTRAIL−) culture used as the negative control. Data from one representative experiment out of five performed are shown. **b** Concentrated supernatants of broth culture of *L. lactis* (hsTRAIL±) bacteria or precipitated proteins from supernatants were subjected to SDS-PAGE electrophoresis and SYPRO^®^ Ruby Protein Gel staining. M—marker; “-”—blank space; Sup.—supernatant; rhTRAIL—recombinant human TRAIL used as positive control; A—hsTRAIL “upper”; B—hsTRAIL “lower” band; PREP.—precipitation using chloroform–methanol method. Data from one representative experiment out of three performed are shown

**Fig. 6:**
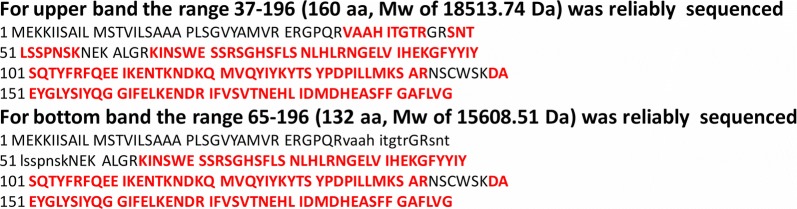
Sequence coverage obtained for upper and bottom band in LC–MS/MS analysis. Red capital letters depicted amino acid (aa) sequence of peptides detected in upper and bottom TRAIL bands, black capital letters depicted aa sequence of peptides not detected in the both TRAIL bands, and black small letters depicted aa sequence of peptides detected only once in the bottom TRAIL band (Table 2). The upper and bottom TRAIL bands were obtained after SDS-PAGE analysis of TRAIL secreted by *L. lactis* (TRAIL+) bacteria

In the next step, using Western blot we confirmed the presence of hsTRAIL in the supernatant from *L. lactis* (hsTRAIL+) broth culture. TRAIL secretion was detected already during the first hour upon induction with nisin, being most effective after 3–4 h (Fig. 5a). Surprisingly, Western blot results revealed that the TRAIL protein appeared in the form of two separate bands and those remained intact for at least 4 h. The subsequent amino acid sequence-mass spectrometry sequencing of protein content in the upper and bottom TRAIL bands, revealed that in both bands TRAIL protein occurs beyond any doubt (Table 1) and in both TRAIL fractions the usp45 leader peptide (27 aa) was cut off. Furthermore, in the upper TRAIL band, additional 9 amino acids from the N-terminus were also cleaved to form the TRAIL molecule consisting finally of 160 aa. In the bottom band only one peptide-spectrum match (PSM) in the sequence range of 37–45 aa and 48–57 aa were identified in comparison to 4 and 9 PSMs measured respectively for upper band (Table 2). Identification of a single PMS can result from a contamination/streaking during electrophoretic separation or the 37–57 aa sequence was detected only in the small fraction of protein molecules present in bottom band. Molecular weights of 18,513 Da and 15,608 Da corresponding to measured fragments of hsTRAIL for upper and bottom band, respectively (Fig. 6), are in good agreement with masses observed in SDS-PAGE electrophoresis (Fig. 5b). It seems that in the bottom TRAIL band, 37 amino acids were cut off from the N-terminus of the TRAIL polypeptide to form a more truncated form, consisting of 132 aa.

**Table 1:** Parameters describing quality of hsTRAIL identification in LC–MS/MS experiments

Description	Score^a^	Coverage^b^	# Peptides^c^	# PSMs^d^
Upper band	10,405.62	73.98	17	372
Bottom band	9530.10	73.98	16	343

^a^Protein scores are derived from ions scores as a non-probabilistic basis for ranking protein hits

^b^Percentage of the sequence of the identified protein covered by the peptides matched

^c^Number of sequenced peptides matched to the protein

^d^Number of peptide-spectrum matches (PSMs)

**Table 2:** List of peptides sequenced in LC–MS/MS experiments for upper and bottom band

Start–end	Sequence	Modifications	# Missed cleavages	Upper band		Bottom band	
				# PSMs	Ion score^a^	# PSMs	Ion score^a^
37–45	*VAAHITGTR*		*0*	*9*	*40*	*1*	*25*
48–57	*SNTLSSPNSK*		*0*	*4*	*39*	*1*	*38*
65–73	KINSWESSR		1	28	64	26	64
66–73	INSWESSR		0	3	46	3	46
74–85	SGHSFLSNLHLR		0	105	69	85	64
86–94	NGELVIHEK		0	22	63	20	63
95–106	GFYYIYSQTYFR		0	16	52	15	66
95–112	FQEEIK		0	2	24	2	24
107–116	FQEEIKENTK		1	47	59	45	57
113–127	NDKQmVQYIYK	M5 (oxidation)	1	31	63	5	65
117–127	NDKQMVQYIYK		1	1	49	25	63
120–127	QmVQYIYK	M2 (oxidation)	0	22	47	21	48
120–127	QMVQYIYK		0	5	44	5	44
120–139	QmVQYIYKYTSYPDPILLmK	M2 (oxidation); M19 (oxidation)	1	1	40	0	0
128–139	YTSYPDPILLmK	M11 (oxidation)	0	41	53	43	54
128–139	YTSYPDPILLMK		0	5	44	12	45
128–142	YTSYPDPILLmKSAR	M11 (oxidation)	1	1	38	3	33
143–166	DAEYGLYSIYQGGIFELK		0	20	78	20	84
143–170	DAEYGLYSIYQGGIFELKENDR		1	7	92	9	105
171–196	IFVSVTNEHLIDmDHEASFFGAFLVG	M13 (oxidation)	0	2	30	2	46

^a^Ions score is − 10*Log(P) where p is the probability that the observed peptide match is a random event

Peptides identified in bottom band based on a single peptide-spectrum match (PMS) are marked in italics

### Aprotynin does not affect proteolysis of secreted hsTRAIL

**Fig. 7:**
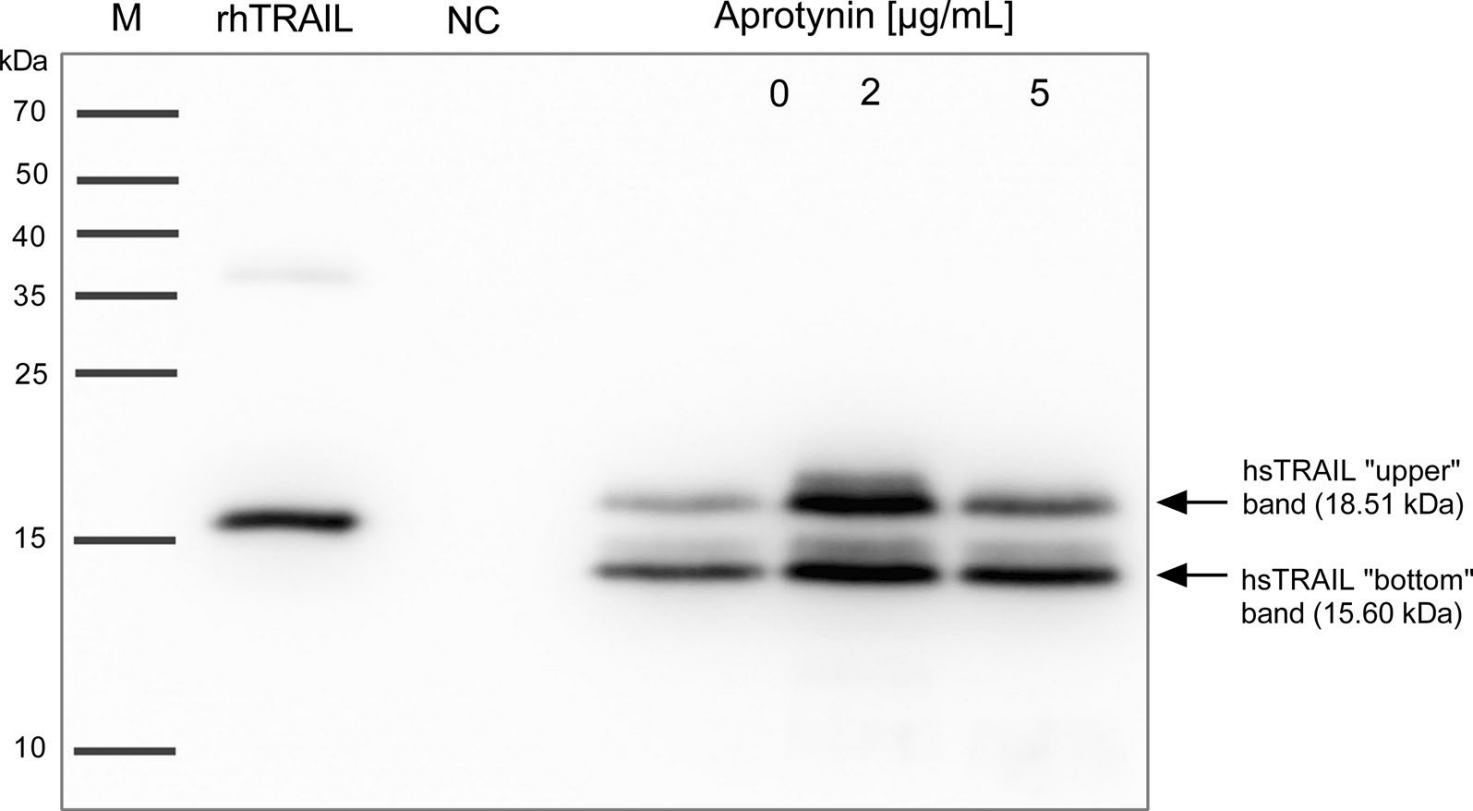
Effect of aprotynin on hsTRAIL secretion by *L. lactis* (hsTRAIL+). The presence of aprotynin during induction of hsTRAIL expression and secretion did not affect proteolysis of secreted hsTRAIL as detected by Western blot. M—molecular weight marker; rhTRAIL (PeproTech)—positive control; NC—protein sample precipitated from supernatant of *L. lactis* (hsTRAIL−) culture as the negative control; 0; 2 and 5 (µg/ml)—concentrations of aprotynin used during induction of protein expression with nisin (25 ng/ml). Data from one representative experiment out of five performed are shown

Heterologous proteins produced by bacterial expression systems are exposed for cleavage by extracellular proteases. The most common proteases in *L. lactis* bacteria, which might affect the secretion of recombinant proteins are serine proteases [18–21]. Therefore, in the next set of experiments, to check if the two forms of our hsTRAIL could be a result of the action of bacterial extracellular proteases, we initiated the expression and secretion of TRAIL in the presence of aprotynin—serine proteases inhibitor. We observed, that addition of aprotynin influenced only the intensity of the bands corresponding to the two forms of TRAIL, but did not affect their presence (Fig. 7). Since the signals for both TRAIL bands were most intensive when aprotynin was used at the concentration of 2 µg/ml, this was selected for further studies.

### hsTRAIL is efficiently secreted to the broth culture supernatant by *L. lactis* (hsTRAIL+)

**Fig. 8:**
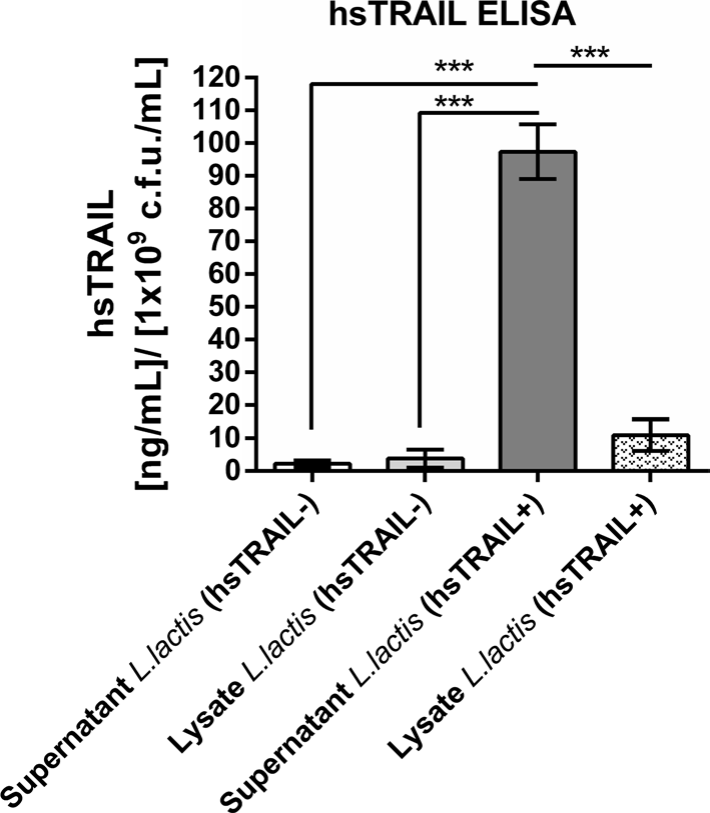
Quantitative determination of hsTRAIL secretion by *L. lactis* (hsTRAIL+) and *L. lactis* (hsTRAIL−) bacteria. Supernatants from broth culture of *L. lactis* (hsTRAIL±) bacteria or corresponding lysates of bacteria were subjected to ELISA to assess the protein secretion efficacy. The bars indicate the mean value ± SD of three independent experiments, each performed in triplicates. Differences between the groups were calculated using two-way ANOVA with Tukey’s multiple comparisons post-test. *p < 0.001

One of the major advantage of NICE expression system is secretion of recombinant protein into the medium. To calculate the efficacy of hsTRAIL secretion, when pNZ8124 plasmid vector with sequence for usp45 leader peptide was used, we performed ELISA and quantified the concentration of the protein both in crude supernatants and cell lysates after 4 h upon the nisin-induction. The hsTRAIL was mostly presented in supernatants from broth culture of *L. lactis* (hsTRAIL+), where achieved the mean concentration of 97.4 ng/ml (Fig. 8). For corresponding cell lysates, the mean hsTRAIL concentration achieved 10.97 ng/ml, indicating the mean secretion efficacy at 89.87% (± SD 95.34; 84.41). The concentration of hsTRAIL in supernatants and lysates from negative control was negligible. Further use of the protein centrifugal concentrators enabled efficient diafiltration of crude supernatant from *L. lactis* (hsTRAIL+) broth culture and concentration of TRAIL sample 10- to 20-fold.

### hsTRAIL produced by *L. lactis* (hsTRAIL+) retains its biological activity and induces apoptosis of human colon cancer cells in vitro

**Fig. 9:**
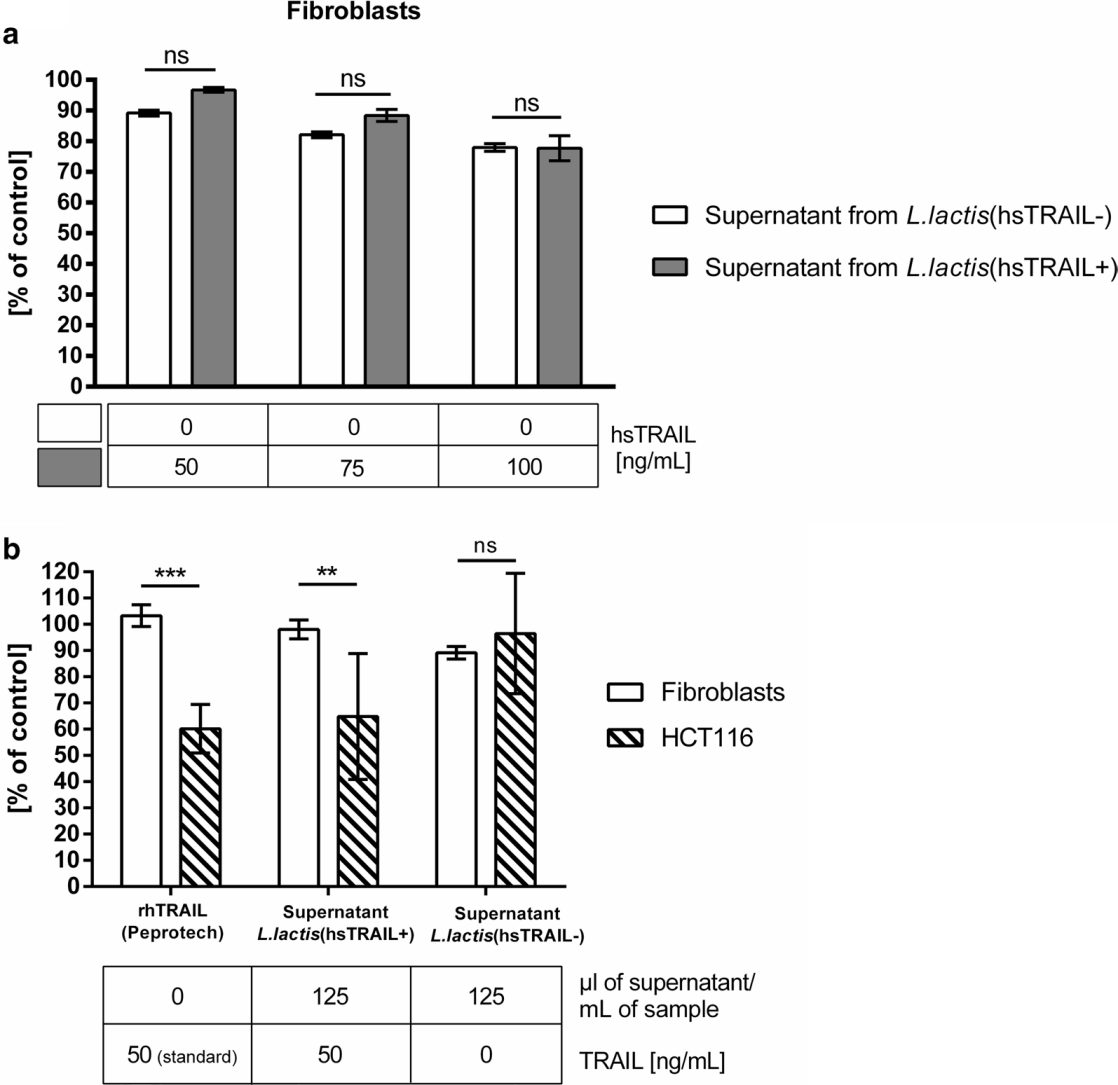
Selectivity of hsTRAIL-induced death to cancer cells. HCT116 cells and human cardiac fibroblasts were incubated for 48 h with increasing concentration of hsTRAIL present in supernatant from broth culture of *L. lactis* (hsTRAIL+). As controls in experimental setup were used: corresponding volumes of supernatants from broth culture of *L. lactis* (hsTRAIL−)—negative control; corresponding concentrations of recombinant human TRAIL (Peprotech)—positive control. Viability of cancer cells and non-malignant, cardiac fibroblasts was assessed by MTS assay. Results are presented as % of viability of cells incubated in standard culture medium only. Secreted hsTRAIL remained non-cytotoxic to fibroblasts (Fig. 9a), while decreased viability of cancer cells in a dose-dependent manner (Fig. 9b). Table below—concentration of hsTRAIL [ng/ml] in specified volume of supernatant. The bars indicate the mean value ± SD of three independent experiments, each performed in triplicates. Statistical significance was calculated using two-way ANOVA with Tukey’s multiple comparison post-test. *p < 0.05, **p < 0.01, ***p < 0.001

**Fig. 10:**
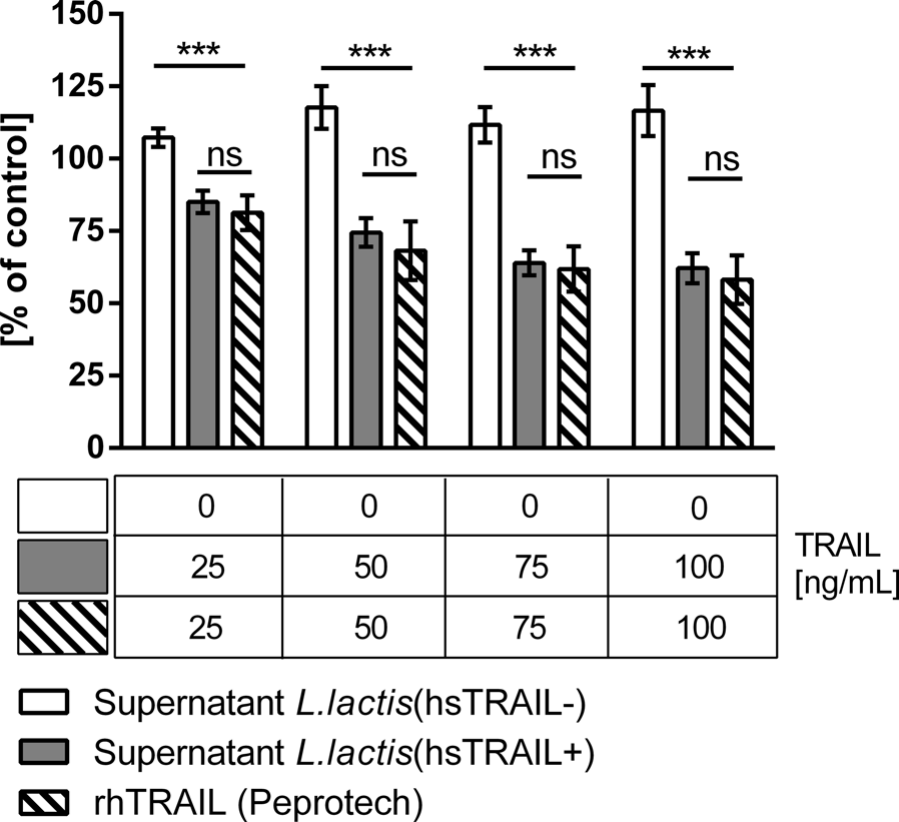
*Lactococcus lactis*-derived hsTRAIL-induced death of human colon cancer HCT116 cells in vitro. HCT116 cells were incubated for 48 h with increasing concentrations of hsTRAIL in supernatants from broth culture of *L. lactis* (hsTRAIL+). The following controls in experimental setup were used: corresponding volumes of supernatants of *L. lactis* (hsTRAIL−)—negative control; corresponding concentrations of recombinant human TRAIL (Peprotech)—positive control. Viability of cancer cells was assessed by MTS assay. Results are presented as % of viability of cells incubated in standard culture medium only. hsTRAIL present in supernatant from *L. lactis* (hsTRAIL+) broth culture decreased viability of cancer cells in a dose-dependent manner, comparable to corresponding concentrations of positive control. Table below—concentration of TRAIL [ng/ml] in specified volume of supernatant or concentration of standard TRAIL formulation (rhTRAIL, Peprotech). The bars indicate the mean value ± SD of three independent experiments, each performed in triplicates. Statistical significance was calculated using two-way ANOVA with Tukey’s multiple comparison post-test. *p < 0.05, **p < 0.01, ***p < 0.001

**Fig. 11:**
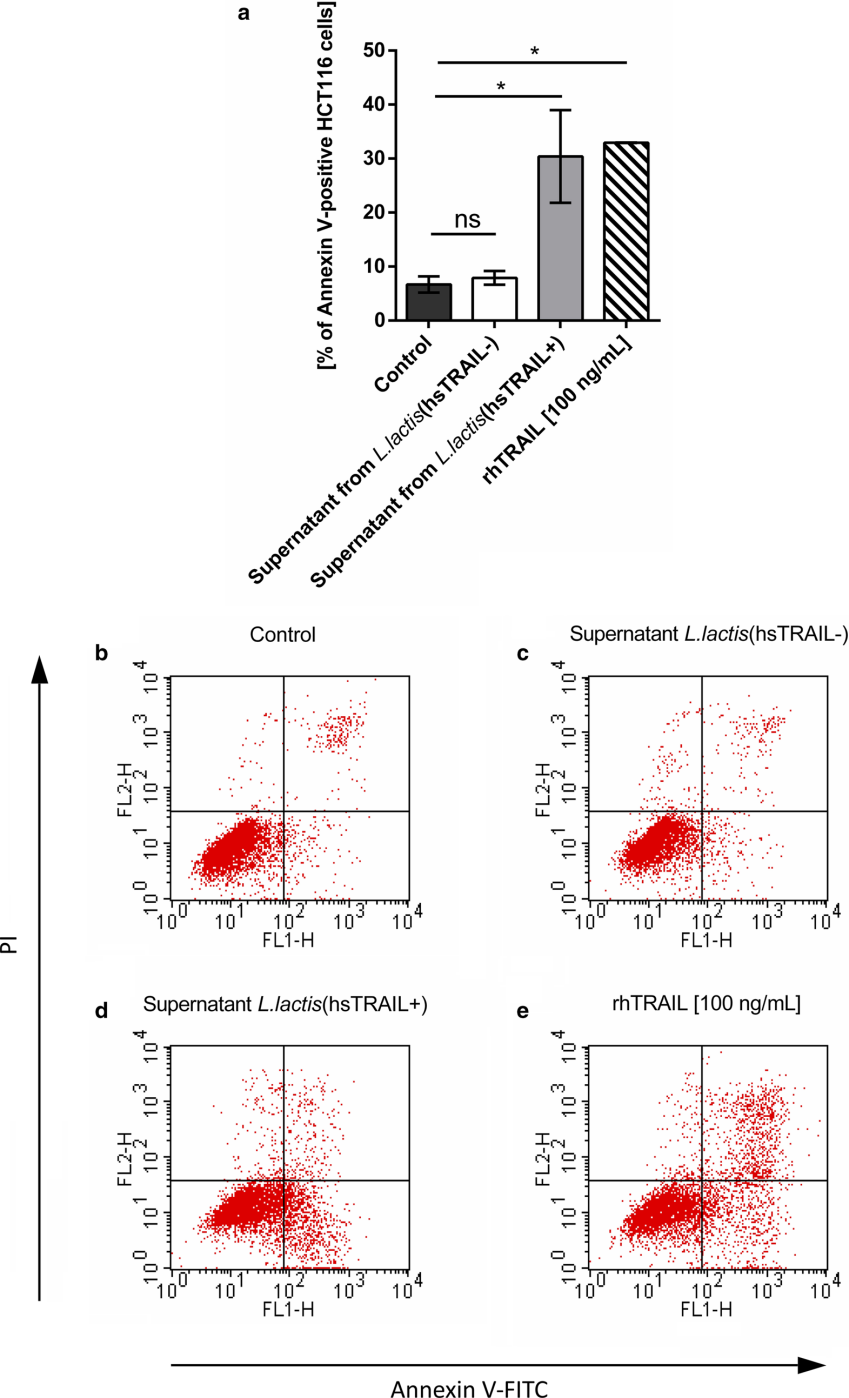
Induction of apoptosis of colon cancer HCT116 cells by hsTRAIL from supernatant of *L. lactis* (hsTRAIL+). HCT116 cells were treated with supernatants from broth cultures of *L. lactis* (hsTRAIL±) bacteria and rhTRAIL (positive control) in a corresponding dose of 100 ng/ml for 48 h and then stained with FITC-Annexin V and PI to determine cancer cells apoptosis. HCT116 turned apoptotic after treatment with supernatant of *L. lactis* (hsTRAIL+) bacteria (**a**, **d**) in contrast to supernatant of *L. lactis* (hsTRAIL−) (negative control; **c**) or medium (**b**). As positive control, standard TRAIL formulation (rhTRAIL, Peprotech) in corresponding concentration was used (**e**). Dot-plots. FL1-H—FITC; FL2-H—PI. The bars (**a**) indicate the mean value ± SD of three independent experiments, each performed in triplicates. Differences between the groups were calculated using two-way ANOVA with Tukey’s multiple comparisons post-test. *p < 0.05

To assess, if hsTRAIL present in broth supernatants from *L. lactis* (hsTRAIL+) culture is biologically active and possess antitumor activity in vitro, we used human colon cancer HCT116 cells incubated with increasing concentrations of *L. lactis* derived-hsTRAIL. Human cardiac fibroblasts, as representatives of rapidly proliferating normal cells were used as a control in parallel. As a positive control, commercially available recombinant TRAIL was used at the same range of concentrations. Cancer and normal cells were incubated either with broth culture supernatant or standard TRAIL preparation for 48 h and their viability was evaluated by MTS assay, which determines activity of mitochondrial enzyme—succinate dehydrogenase. In living cells, this enzyme converts yellow tetrazole (MTS) into blue formazan, therefore the absorbance signal is proportional to the number of living cells. The obtained results (Fig. 9a) document that in contrast to fibroblasts, for which hsTRAIL did not show any cytotoxicity, a decreasing viability of cancer cells, alongside with increasing concentration of the produced hsTRAIL, was detected. In keeping, control supernatant from the broth culture of *L. lactis* mock transfected, harbouring a plasmid vector without TRAIL-cDNA insert—*L. lactis* (hsTRAIL−) did not affect viability of colon cancer HCT116 cells (Figs. 9b, 10). These results proved, that hsTRAIL, produced by selected clone of *L. lactis* bacteria, remains biologically active and selectively kills colon cancer cells in vitro. Moreover, comparison of the antitumor activity of hsTRAIL from the supernatant of *L. lactis* (hsTRAIL+) culture, with the same concentrations of TRAIL, as of the commercially available human TRAIL preparation has shown that our hsTRAIL possesses the same biological activity against tumor cells as the standard (Fig. 10). Since TRAIL eliminates cancer cells via extrinsic apoptotic pathway, we examined the effect of *L. lactis*-derived hsTRAIL on HCT116 apoptosis (Fig. 11). Flow cytometry analysis showed that 30% of HCT116 cells were apoptotic after treatment with supernatant from *L. lactis* (hsTRAIL+) bacteria, similarly to positive control (Fig. 11a, e), in contrast to non-treated cells (Fig. 11a, b) or cells incubated with supernatant from *L. lactis* (hsTRAIL−) (Fig. 11a, c).

## Discussion

The use of TRAIL in the clinic is of great hope because of its selective action against malignant cells. Another potential clinical advantage of TRAIL is its synergism with some chemotherapeutics currently in use, which may enable decreasing of their dose, thus reducing side effects. This might improve not only prognosis, but also patients’ quality of life. Therefore, the current research is focusing on new TRAIL-formulation and delivery strategies, helping to overcome the problem with its short biological half-life in humans. Part of these new strategies represent, among others, bacterial expression systems. In 2010, Zhang et al. showed that tumor-bearing mice injected intravenously with *E. coli* producing human recombinant TRAIL, localized and replicated in tumor tissues of murine B16 melanoma, its metastases and human H460 lung carcinoma [22]. The observed bioactivity of TRAIL and tumor tropism of *E. coli* might classify this model as ideal for specific tumor targeting immunotherapy. However, when using *E. coli* for delivery of therapeutics in vivo, there is still a risk of complications, including bacteremia or even endotoxic-shock. Problem of the formation of insoluble inclusion bodies during overexpression of recombinant TRAIL by *E. coli* has been also shown in some recent studies [23, 24]. Another examined bacterial vector for TRAIL was attenuated *Salmonella typhimurium*, which besides production of bioactive protein, has shown activation of the immune system of BALB/c nude mice [25]. However, *S. typhimurium* is mainly intracellular bacterium, thus might reduce the efficiency of the produced TRAIL acting with specific membrane receptors on target cells.

*Lactococcus lactis* NZ9000 strain has been used for production of many biologically active proteins, assuming its potential use in future therapies, e.g. IL-12—in the treatment of asthma [26], IL-10 and TGF-β1—inflammatory bowel disease (IBD) [27–29], insulin-like growth factor I (IGF-I)—colitis [30], recombinant mouse heme oxygenase-1 (rmHO-1) [31], and many others [32–34]. In cancer therapy, the potential use of *L. lactis* NZ9000 strain concerned breast cancer, namely hemagglutinin-neuraminidase (HN) protein of Newcastle disease virus (NDV) [35].

In our study we have documented the expression and secretion of bioactive human TRAIL by *L. lactis* NZ9000 strain harbouring the plasmid containing cDNA for codon-optimized human soluble TRAIL. The step of codon-optimization was relevant since the codon usage is a key factor in assessing probability of successful and efficient expression of heterologous genes in *L. lactis* [15]. After selection of hsTRAIL-expressing clone, we optimized conditions for the culture of *L. lactis* (hsTRAIL+) strain. Our results showed that cell density at the time of induction and the concentration of nisin added as the inducer, are most critical parameters. In the culture of lactic acid bacteria (LAB), the lactic acid fermentation is a dominant metabolic process of sugar conversion into cellular energy. The main, final fermentation product of this conversion is lactic acid which is toxic [36]. However, since the toxicity of lactic acid depends on its non-dissociated form, increasing of pH of the medium for bacterial culture allows to elongate the period of their growth. We showed, that supplementation of standard culture medium for *L. lactis* (hsTRAIL+) bacteria with l-arginine, as additional source of energy, enables the maintenance of neutral pH. An efficient reduction of acidity and improvement of bacterial biomass, after arginine supplementation, has been shown recently by Laroute et al. [37]. This “buffering” effect of arginine results from the formation of ammonium ion in the route of arginine deiminase pathway [37]. Another important issue related to pH level of cell culture is ATR effect (acid tolerance response) [20], which suppression has been shown to improve the production level and stability of secreted recombinant proteins [20, 38]. We showed that sufficiently high concentration of glucose, besides l-arginine, is also important, and only the combination of these two sources of energy in concentrations ≥ 0.3%, resulted in an optimal density of bacterial cell growth. The concentration of nisin, the inducer, as the second parameter [17] used for recombinant protein expression (NICE system), was established at 25 ng/ml, which occurred to be the lowest effective dose.

We observed that *L. lactis* (hsTRAIL+) strain produces human TRAIL in two separate forms, suggesting its enzymatic cleavage by bacterial proteases. The *L. lactis* proteolytic system involves two major proteases: intracellular ClpP and an extracellular trypsin-like serine protease HtrA [28]. The standard role of HtrA is degradation of exported abnormal proteins. In the study performed by Poquet et al. [28], also working with the NICE system and *L. lactis* NZ9000 strain, two novel actions of this proteinase were observed and described, namely a pro-peptide processing of the native host proteins and their maturation [18]. In further studies, the same authors described the role of HtrA proteinase in proteolysis of secreted recombinant proteins concluding, that the mutation of htrA gene leads to the stabilization of recombinant proteins [19, 21]. From the other hand, Sriraman et al. [20], attributed the role of HtrA in secretion of recombinant proteins with the mechanism of induction, because of HtrA involvement in alternations of bacterial cell wall permeability properties. Moreover, mutation of htrA gene increased aggregation of cells and therefore reduced membrane exposure for inducer, e.g. nisin, affecting activation of genes involved in a secretion machinery [20]. To check, whether the observed pattern was a result of action of *lactococcal* extracellular proteases, we initiated the expression and secretion of TRAIL in the presence of serine proteases inhibitor, aprotynin. However, the addition of aprotynin to the culture broth, had no effect on the appearance of the two forms of hsTRAIL, suggesting that bacterial extracellular proteases are not involved in the cleavage of hsTRAIL. It is worth mentioning that the pattern of two bands for hsTRAIL secreted by *L. lactis* (hsTRAIL+) strain has been detected in every single Western blot, even if the native (without protein precipitation) *L. lactis* (hsTRAIL+) culture broth supernatant was electrophoresed (Fig. 5b). Analysis of the amino acid sequences performed on the two hsTRAIL protein bands showed that the upper (larger) band represents a slightly truncated form of hsTRAIL containing 160 amino acid (aa) residues, while the lower (smaller) band represents a more truncated form of hsTRAIL deprived of 37 aa residues from the N-terminus of the protein, both having the bacterial usp45 leading peptide cut off. It is also worth noting that the positive control (rhTRAIL, Peprotech), was a protein encoded by the *E. coli* strain and the folding nature of hsTRAIL expressed by *L. lactis* could be quite different, therefore, vertical positioning of these proteins on the Western blot may not indicate their exact molecular size. However, analysis of the mechanism of post-translational modifications of hsTRAIL in *L. lactis* bacteria goes beyond the scope of the current paper.

To assess the in vitro cytotoxicity of hsTRAIL we incubated colon cancer cells with supernatants from *L. lactis* (hsTRAIL+) bacteria. In parallel, normal human fibroblasts were incubated in the same conditions. Using MTS assay to assess cell viability, we observed that hsTRAIL produced by recombinant *L. lactis* (hsTRAIL+) bacteria efficiently and selectively induced apoptosis of cancer cells in a dose-dependent manner. When comparing antitumor activity of purified and concentrated hsTRAIL, obtained from broth culture of nisin-induced *L. lactis* (TRAIL+) with commercial TRAIL probe at the same concentration range, we showed almost the same level of TRAIL-mediated cytotoxicity against HCT116 human colorectal cell line and confirmed the mechanism as apoptosis. However, determination of the precise biological activity of these two TRAIL protein requires further investigations.

## Conclusions

In conclusion, our findings document the culture and expression conditions enabling production of human soluble TRAIL by recombinant *L. lactis* strain, which effectively eliminates HCT116 human colon cancer cells via apoptosis in vitro. Using *L. lactis* bacteria, as a live vector for TRAIL delivery for a potential future treatment may have many advantages, e.g. production of proteins by non-pathogenic bacteria and local effect of secreted therapeutic protein. Further studies using in vivo models in human colorectal cancer-bearing mice have been already undertaken to provide evidence if the hsTRAIL-expressing *L. lactis* bacteria could be used as an effective and safe producer of TRAIL for future clinical use.

## Methods

### Cell cultures

Human colon carcinoma cell line HCT116 was obtained from the American Type Culture Collection (ATCC, Manassas, VA) and maintained according to the ATCC’s instructions. Human primary proliferating cardiac fibroblasts were obtained from Cell Applications, Inc. (San Diego, CA). Briefly, HCT116 cells were cultured in McCoy’s 5A medium, supplemented with 10% fetal bovine serum (FBS) and gentamicin (50 µg/ml) (all from Gibco, Paisley, UK) in a 37 °C humidified atmosphere with 5% CO_2_. Human cardiac fibroblasts were cultured in Dulbecco’s Modified Eagle’s Medium (DMEM) (Sigma Aldrich, Saint Louis, MI) supplemented with 10% FBS and 1:100 penicillin/streptomycin (all from Life Technologies, Carlsbad, CA) in a 37 °C humidified atmosphere with 5% CO_2_. The cells were regularly tested for *Mycoplasma* sp. contamination by PCR-ELISA kit (Roche, Mannheim, Germany) and for endotoxin contamination by the Limulus test (Charles River Laboratories, Wilmington, MA) according to manufacturer’s instruction.

### Bacterial cell cultures

*Lactococcus lactis* NZ9000 host strain, a derivate of *L. lactis* subsp. *cremoris* MG1363 with regulatory genes (nisR, nisK) integrated into the pepN gene of MG1363 [15], was obtained from MoBiTec (Goettingen, Germany) and cultured in M17 medium (BTL, Lodz, Poland) supplemented with 0.5% glucose (POCH, Gliwice, Poland). For culture of *L. lactis* clones harbouring secretion plasmid vector pNZ8124 (MoBiTec) and its modified derivatives with the human soluble TRAIL-cDNA placed downstream of the inducible promoter PnisA on the plasmid pNZ8124, chloramphenicol (10 µg/ml; Sigma Aldrich) was added to maintain the plasmid.

### hsTRAIL-cDNA cloning

A synthetic human soluble TRAIL-cDNA (hsTRAIL-cDNA) with optimized codons was designed to fit the codon usage pattern (codon bias) of the *L. lactis* host. This synthetic cDNA construct contained 169 codons plus stop codon, including 76 original and 94 changed codons for those more frequently found in *L. lactis* highly expressed genes. Synthetic hsTRAIL-cDNA construct was commercially synthetized by Eurofins Genomic (Ebersberg, Germany). Then, constructed hsTRAIL-cDNA sequence was ligated, using T4 DNA ligase (EurX, Gdansk, Poland), to EcoRV and XbaI—linearized plasmid vector pN8124 (MobiTec), containing sequence coding for signal peptide of lactococcal usp45 gene. Prepared pNZ8124-hsTRAIL plasmid vector was transformed into the electrocompetent *L. lactis* NZ9000 host strain cells by electroporation, using Gene PulserXcell™ Electroporation System (BioRad, Hercules, CA), according to vector producer’s instruction (MoBiTec).

### Selection of a positive clone of *L. lactis* harbouring recombinant plasmid pNZ8124-hsTRAIL

Positive clone of *L. lactis* containing insert for hsTRAIL-cDNA after electroporation with plasmid vector pNZ8124-hsTRAIL was selected in two-steps. First, isolated plasmids were cleaved with *Eco*RV and *Xba*I restriction enzymes (EurX) and the presence of hsTRAIL-cDNA insert was defined by the size of cleaved fragments using agarose gel electrophoresis (1.5% agarose gel). In the second step, selected fragments were purified from the gel and amplified by PCR method using the following primers: 5′-TGGTACTCGTGGTCGTAGCA-3′ sense and 5′-GAAGCTTCGTGGTCCATGTC-3′ antisense (Genomed, Warsaw, Poland). Clone number 3 was selected as TRAIL-positive, designated as *L. lactis* (hsTRAIL+) and used for further studies.

### Optimization of culture conditions for the recombinant *L. lactis* (hsTRAIL+) clone

M17 broth medium (BD Difco, Franklin Lakes, NJ) supplemented with 0.5% glucose (Gluc) and 10 µg/ml of chloramphenicol (Cm10), was inoculated with *L. lactis* (hsTRAIL+) glycerol stock and grown overnight at 30 °C, without aeration. To optimize conditions for culture of the recombinant *L. lactis* clone, the following culture media were prepared: M17 supplemented with 0.5% Gluc, Cm10; M17 supplemented with 0.1% Gluc, 0.1% of l-arginine (Arg) and Cm10; M17 supplemented with 0.3% Gluc, 0.3% Arg and Cm10, and then were inoculated with an overnight pre-culture of bacteria in a dilution of 1:20, and incubated at 30 °C, without aeration. The OD_600_ and pH of the cell cultures were determined after 4 and 24 h. A 4-h culture period was selected and further used for the production of hsTRAIL by *L. lactis* producer (clone no. 3) upon induction with nisin.

### Induction of hsTRAIL expression with nisin

For induction of hsTRAIL expression and secretion by *L. lactis* (hsTRAIL+) bacteria, the M17 broth medium supplemented with 0.5% Gluc and Cm10 was inoculated with *L. lactis* (hsTRAIL+) or *L. lactis* with empty vector pNZ8124—*L. lactis* (hsTRAIL−)—used as a negative control. Cultures were diluted 1:40 and grown overnight at 30 °C, without aeration. Overnight cultures were diluted 1:20 in M17 broth medium supplemented with 0.3% Gluc, 0.3% Arg, Cm10 and ZnSO_4_ (100 µM) and grown for additional 3 h at 30 °C without aeration until OD_600_ = 0.3–0.4. After incubation, the cultures were centrifuged for 30 min at 2800×*g* at room temperature and the cell pellets were resuspended in 1/4 volume of M17 supplemented with 0.3% Gluc, 0.3% Arg, Cm10, ZnSO_4_ (100 µM) and aprotynin (BioShop, Burlington, Canada), as the serine proteases inhibitor, to prevent potential cleavage of expressed hsTRAIL protein, and were induced for hsTRAIL expression with nisin (MoBiTec). For optimization of induction conditions, the cultures were induced with the following concentrations of nisin: 10; 25; 35; 50; 80 ng/ml in M17 medium supplemented as above. The optimal concentration of aprotynin was established experimentally from 2 to 5 µg/ml tested. After 4 h of incubation, the OD_600_ of the cultures was measured to monitor the bacterial growth and then the cells were centrifuged for 30 min at 2800×*g* at 4 °C. Cell-free supernatants were collected, pH was measured and neutralized (if necessary) to pH = 7 with NaOH (POCH, Gliwice, Poland). TRAIL samples concentration was performed using disposable The Thermo Scientific™ Pierce™ PES 10 K protein concentrators (Pierce Biotechnology, Rockford, IL) for centrifugal ultrafiltration, according to the manufacturer’s instructions. Concentrated supernatants were filtered through low protein-binding filters (Merck-Millipore, Burlington, MA), aliquoted and stored at − 80 °C until use for further studies.

### Isolation of hsTRAIL protein

hsTRAIL was precipitated as total protein content from sterile culture supernatants using chloroform–methanol protein extraction method [39]. Briefly, 600 µl of methanol was added to 150 µl of culture supernatant and vortexed, after which chloroform, at ratio 1:1 to the starting volume of the supernatant, was added and vortexed again. Next, 450 µl of H_2_O was added, and the whole suspension was vortexed and centrifuged for 5 min at 14,000×*g*. The top, aqueous layer was removed, then 600 µl of methanol was added and the mixture was vortexed and centrifuged for 10 min at 14,000×*g*. Methanol was removed and the pellet was dried under vacuum for 1.5 h, and resuspended in Bacterial Protein Extraction Reagent (BPER, Thermo Fisher Scientific, Waltham, MA) with addition of protease inhibitor cocktail (Pierce, Waltham, MA) and stored at − 20 °C until further use.

### Western blot analysis of hsTRAIL produced by *L. lactis* (hsTRAIL+) bacteria

The protein samples precipitated from supernatants of *L. lactis* (hsTRAIL±) cultures were used for protein separation by the SDS-PAGE electrophoresis and Western blot analysis. Protein concentration was measured using BCA (Bicinchoninic acid) protein assay kit (Pierce) and the equal amounts of the samples were mixed with LDS sample buffer (lithium dodecyl sulfate at pH of 8.4, Invitrogen, Carlsbad, CA) and reducing buffer (50 mM dithiothreitol—DTT; Invitrogen), incubated at 70 °C for 10 min and loaded onto 14% SDS-PAGE gel. Recombinant human TRAIL (rhTRAIL; PeproTech, London, UK) was used as a positive control. After electrophoresis, separated proteins were transferred onto the polyvinylidene fluoride membrane (PVDF, BioRad) using Trans-Blot Turbo Transfer System (BioRad). Subsequently blots were blocked for 1 h at room temperature with 5% of nonfat milk in TTBS buffer (50 mM Tris–HCl, pH 7.6; 150 mM NaCl, 1% Tween-20). The protein bands were detected using the following antibodies: primary—mouse anti-human sTRAIL/Apo2L monoclonal antibodies (dilution 1:1000; Santa Cruz Biotechnology, Santa Cruz, CA); secondary—goat anti-mouse HRP-conjugated IgG (dilution 1:8000; Santa Cruz Biotechnology) and visualized with the SuperSignal West Pico Chemiluminescence Substrate kit (Pierce) according to the manufacturer’s protocol and analysed with KODAK GEL LOGIC 1500 Digital Imaging System (KODAK, Rochester, NY).

### SYPRO^®^ Ruby Protein gel staining

The protein samples precipitated from supernatants of *L. lactis* (hsTRAIL±) cultures, or crude supernatants samples, were used for protein separation by the SDS-PAGE electrophoresis. Protein concentration was measured using BCA (Bicinchoninic acid) protein assay kit (Pierce) and the equal amounts of the samples were mixed with LDS sample buffer (lithium dodecyl sulfate at pH of 8.4, Invitrogen, Carlsbad, CA) and reducing buffer (50 mM dithiothreitol—DTT; Invitrogen), incubated at 70 °C for 10 min and loaded onto 14% SDS-PAGE gel. After electrophoresis, SYPRO^®^ Ruby Protein gel staining (Molecular Probes, Eugene, US) was performed to detect proteins present in supernatants. The gel was fixed (50% methanol, 7% acetic acid) for 30 min, stained overnight with SYPRO^®^ Ruby gel stain, washed in wash solution (10% methanol, 7% acetic acid) for 30 min. and rinse twice in ultrapure water. The gel was analyzed using ChemiDoc™ Imaging system (BioRad).

### Sequencing TRAIL protein by mass spectrometry (LC–MS/MS)

The visualized SDS-PAGE bands (Coomassie Brilliant Blue staining) were cut out and proteins were reduced, alkylated, and digested according to the protocol described previously [40]. Peptides were analyzed with the use of a Q-Exactive mass spectrometer (Thermo Fisher Scientific) coupled with nano-HPLC (UltiMate 3000 RSLCnano System, Thermo Fisher Scientific) as previously described with minor modifications [41]. Peptides were separated using a 90 min gradient of acetonitrile from 2 to 40% in the presence of 0.05% formic acid. The Top 8 method was used for mass spectrometry measurement with full MS and MS/MS resolution of 70,000 and 35,000 respectively. Database searching of RAW files was performed in Proteome Discoverer 1.4 (Thermo Fisher Scientific) MASCOT 2.5.1 (Matrix Science Ltd, London, UK) was used for database searching against the common Repository of Adventitious Proteins (cRAP) database containing the sequences of recombinant tumor necrosis factor ligand superfamily member 10 (TRAIL), *lactococcal* protein usp45 and common contaminants. The following search parameters were applied: enzyme specificity—trypsin; permitted number of missed cleavages—1; fixed modification—carbamidomethylation (C); variable modifications—oxidation (M), deamidation (NQ); precursor mass tolerance—± 10 ppm; fragment mass tolerance—± 20 mmu. Identifications with a score value over 80 were accepted.

### Assessment of TRAIL secretion efficacy

Induction of hsTRAIL secretion was performed as described above. The *L. lactis* cells in broth culture were centrifuged for 30 min at 2800×*g* at 4 °C and extraction of protein from bacterial cells pellet was performed. The cells were resuspended in Bacterial Protein Extraction Reagent (BPER, Thermo Fisher Scientific) with addition of protease inhibitor cocktail (Pierce), 10 U of DNase-I and 5.6 mg/ml of lysozyme (both from Thermo Fisher Scientific), then incubated for 1 h at 37 °C and 10 min. at 70 °C to inhibit the enzymes. After centrifugation (10 min., 15,000×*g*, RT) cell lysates were collected and stored at − 80 °C until further use. To assess the concentration of hsTRAIL in the lysate from recombinant *L. lactis* (hsTRAIL+) bacteria and secreted to the broth culture medium during induction, ELISA for human soluble TRAIL (LSBio™, Seattle, WA) was performed according to the manufacturer’s instructions. The absorbance was measured at 450 nm and 570 nm (wavelength correction) using microplate reader ELx 800NB (BIO-TEK INSTRUMENTS, Winooski, VT).

### Cell viability assay

Cytotoxic activity of hsTRAIL from the culture supernatant of *L. lactis* (hsTRAIL+) against human colon cancer HCT116 cells and human cardiac fibroblasts was determined using 3-(4,5-dimethylthiazol-2-yl)-5-(3-carboxymethoxyphenyl)-2-(4-sulfophenyl)-2*H*-tetrazolium (MTS) assay **(**CellTiter 96^®^ AQ_ueous_ One Solution Cell Proliferation Assay, Promega, Madison, WI). Briefly, HCT116 cells and human cardiac fibroblasts were seeded onto flat-bottom 96-well plates (Sarstedt, Numbrecht, Germany) at a density of 10^4^/5 × 10^3^ cells per well in McCoy’s 5A/DMEM medium, respectively, containing 2% FBS. After 20 h for cell attachment, the supernatant from the culture of *L. lactis* (hsTRAIL+) was added to the cells in the dilutions corresponding to the concentrations of hsTRAIL: 25; 50; 75; 100 ng/ml, respectively (measured using ELISA Kit (LSBio™, Seattle, WA). As a negative control, the supernatant from the cultures of *L. lactis* (hsTRAIL−) was added to the cells in corresponding volumes. As a positive control, recombinant human TRAIL (rhTRAIL; PeproTech) was used in the same range of concentrations. After 48 h of incubation 20 µl per well of MTS (CellTiter 96^®^ AQ_ueous_ One Solution Cell Proliferation Assay, Promega) dye solution was added directly into the culture wells and incubated for additional 2 h. The quantity of formazan product, directly proportional to the number of living cells in culture, was detected by absorbance measurement at 490 nm with a 96-well plate reader (Spark^®^ Tecan, Mannedorf, Switzerland).

### Detection of apoptosis by flow cytometry

Apoptosis of HCT116 cells was determined using FITC-Annexin V Apoptosis Detection kit (BD Pharmingen, New Jersey, US) according to the to the manufacturer’s instructions. Briefly, HCT116 cells were seeded onto flat-bottom 24-well plate (Sarstedt) at a density of 10^5^ cells per well in McCoy’s 5A medium containing 2% FBS. After 20 h for cell attachment, the supernatant from the culture of *L. lactis* (hsTRAIL+) was added to the cells in the dilutions corresponding to the concentration of hsTRAIL 100 ng/ml. As a negative control, the supernatant from the cultures of *L. lactis* (hsTRAIL−) was added to the cells in corresponding volume. As a positive control, recombinant human TRAIL (rhTRAIL; PeproTech) was used in the same concentration. After 48 h of incubation, the cells were washed twice in ice-cold PBS, trypsynized and cell pellets were respuspended in 1× Annexin V Binding Buffer (0.01 M Hepes/NaOH (pH 7.4), 0.14 M NaCl, 2,5 mM CaCl_2_). The cells were stained with Annexin V-FITC and PI for 15 min at RT in the dark, followed by FACS analysis using FACSCalibur (Becton–Dickinson Immunocytometry System, Palo Alto, CA) by using CellQuest (version 3.1) software.

### Statistical analysis

Statistical analysis was performed using GraphPad Prism Software version 4.00 (2003). Statistical significance was calculated using two-way ANOVA with Tukey’s multiple comparisons post-test. *p < 0.05, **p < 0.01, ***p < 0.001. The data from each assay are representative for 3–5 independent experiments.
